# Fabry disease and multiple sclerosis misdiagnosis: the role of family history and neurological signs

**DOI:** 10.18632/oncotarget.23970

**Published:** 2018-01-05

**Authors:** Paolo Colomba, Carmela Zizzo, Riccardo Alessandro, Giuseppe Cammarata, Simone Scalia, Antonello Giordano, Maurizio Pieroni, Luigi Sicurella, Luisa Amico, Alessandro Burlina, Giovanni Duro

**Affiliations:** ^1^ National Research Council, Institute of Biomedicine and Molecular Immunology “A. Monroy”, Palermo, Italy; ^2^ Department of Biopathology and Medical Biotechnology, Biology and Genetics Section, University of Palermo, Palermo, Italy; ^3^ Department of Neurology, “R. Guzzardi” Hospital-ASP Ragusa, Vittoria, Italy; ^4^ Cardiovascular Department, St. Donato Hospital, Arezzo, Italy; ^5^ Complex Operative Unit of Neurology, St. Antonio Abate Hospital, Trapani, Italy; ^6^ Unit of Nephrology, Ospedali Riuniti Villa Sofia-Cervello, Palermo, Italy; ^7^ Neurological Unit, St. Bassiano Hospital, Bassano del Grappa, Italy

**Keywords:** fabry disease, multiple sclerosis, misdiagnosis

## Abstract

Fabry disease (FD) is an X-linked inherited lysosomal storage disorder caused by α galactosidase A (α-gal A) deficiency. Central nervous system involvement and chronic white matter lesions are observed in both FD and multiple sclerosis (MS), which can confound the differential diagnosis. We analyzed the *GLA* gene, which encodes α-gal A, in 86 patients with clinical and neuroradiological findings consistent with MS to determine whether they had FD. We identified four women initially diagnosed with MS who had *GLA* mutations associated with FD. Our results indicate that family history besides neurological findings should be evaluated in patients with an uncertain diagnosis of MS. Also the involvement of organs outside the central nervous system can support the FD diagnosis.

## INTRODUCTION

Fabry disease (FD) is a rare lysosomal storage disorder. It is caused by mutations in the *GLA* gene, which encodes the enzyme α-galactosidase A (α-gal A), that result in α-gal A deficiency and the progressive accumulation of globotriaosylceramide and its derivatives in lysosomes [1]. This triggers a cascade of cellular events including in vascular endothelium [2]. The disease usually manifests in childhood or early adolescence with the emergence of angiokeratomas, corneal opacities (cornea verticillata), microalbuminuria and/or proteinuria, and symptoms that reflect the involvement of the autonomic nervous system including neuropathic pain, pain crises, and hypohidrosis [3]. Disease progression is characterized by progressive deterioration of renal function resulting in end-stage renal disease and the development of serious cardiovascular and cerebrovascular complications that can cause premature death [4]. Central nervous system (CNS) manifestations include stroke and cerebrovascular disease (i.e. chronic white matter lesions, CWML) [5]. Since the *GLA* gene is on the X chromosome, women usually present with milder and more variable symptoms compared to men. Therefore, female patients can be more difficult to diagnose.

Because the clinical features of FD overlap with those of other disorders, errors and delays in diagnosis are common [6, 7]. FD can be misdiagnosed as multiple sclerosis (MS) because patients with either disease can present with pain and white matter lesions on magnetic resonance imaging (MRI). Several studies have described FD patients who were initially diagnosed with MS [7–11], or who were later found to have both diseases [12]. For example, Lidove et al. described 58 FD patients, of which four were initially diagnosed with MS [7]. Böttcher *et al*. in a cohort of 187 FD patients identified 11 subjects who were formerly diagnosed with “possible” or “definite” MS [8]. The diagnosis of MS is generally based on clinical manifestations, MRI, and cerebrospinal fluid analysis. However, a fraction of patients diagnosed with MS do not fully meet the diagnostic criteria [13–15]. In this study, we report four patients with FD among a cohort of 86 patients who received a previous “possible” diagnosis of MS.

## RESULTS

We investigated 86 patients (58 female and 28 male; average age of 42 years, range 18–66 years) who had previously received a “possible” diagnosis of MS. All patients presented with nervous system involvement. Brain MRI demonstrated white matter lesions. Four women out of the cohort of 86 patients (4.7%) were found to have mutations in *GLA* that are responsible for FD. The demographic and clinical data for the FD patients are summarized in Table 1.

**Table 1:** Demographic, genetic, biochemical and clinical data of the four patients with Fabry disease

Pat. No	Sex/Age	GLA gene mutations	α-GAL A activity	CWML	CNS involvement	Neuropathic pain	Other signs
1	F/27	c.718_719delAA	2.5	+	+	-	-
2	F/26	M51I	0.0	+	-	+	-
3	F/63	R342Q	4.1	+	-	-	recurrent headache
4	F/45	G395A	6.1	+	+	-	recurrent fever, abdominal pain

CWML, chronic white matter lesions; CNS, central nervous system; +, yes; -, no.

α-GAL A activity is measured in nmol/ml/h (normal values >3).

Patient 1 is a 27-year-old woman with a history of a transient ischemic attack. Her father was diagnosed with FD. He was found to have a previously reported pathogenic variant (c.718_719delAA) in *GLA* [16]. The same mutation was identified in our patient and her α-gal A activity was 2.5 nmol/ml/h, which is slightly below the reference values for healthy subjects (normal values >3 nmol/ml/h).

Patient 2 is a 26-year-old woman who was evaluated for burning pain in the limbs. A brain MRI demonstrated the presence of multiple white matter lesions. She had a mutation (M51I) in *GLA* that resulted in no α-gal A activity, which is uncommon in women with FD. The same mutation was previously identified in seven individuals in her family (five women and two men). The M51I mutation is associated with the atypical form of FD [17, 18]. Variability in organ involvement and disease severity was observed among these individuals, with some found to have low or no α-gal A activity [18].

Patient 3 is a 63-year-old woman who had a recurrent headache. A brain MRI demonstrated white matter lesions suggestive of MS. Genetic analysis of *GLA* gene revealed the presence of the R342Q mutation, which is responsible for the classic form of FD [19]. Normal α-gal A activity was detected (4.1 nmol/ml/h). The same mutation was previously identified in a male cousin of the patient. This individual exhibited the typical manifestations of FD and had no detectable α-gal A activity.

Patient 4 is a 45-year-old woman who experienced a juvenile stroke. She also complained of recurrent fever and abdominal pain. A mutation (G395A) in *GLA* was detected and α-gal A activity was within the normal range (6.1 nmol/ml/h). We previously identified this pathogenic mutation in nine patients with signs and symptoms of FD. It was associated with α-gal A deficiency in male subjects [20]. The same mutation was identified in three other individuals in patient's family who had not been diagnosed with the disease yet.

We identified an additional 43-year-old woman who was initially diagnosed with “possible” MS. However, the diagnosis was not confirmed following clinical work-up. Her symptoms included acroparaesthesia, burning pain in the limbs particularly after physical activity, heat and cold intolerance, recurrent headache, and abdominal pain. Genetic analysis revealed a mutation (S126G) in *GLA.* Her α-gal A activity was within the normal range (3.5 nmol/ml/h). The pathogenic nature of S126G mutation is uncertain [21, 22]. We determined that five other members of her family have the same mutation and are currently under observation.

## DISCUSSION

According to the current diagnostic criteria [13–15], the diagnosis of MS is based on clinical manifestations, cerebral MRI findings, and the presence of oligoclonal bands in cerebrospinal fluid with increased intrathecal IgG synthesis (adjunct criteria). Alternative diagnoses should be excluded [23]. Several diseases can mimic MS leading to difficulties in diagnosis and possible misdiagnosis [24–26]. Approximately 5–10% of patients received a misdiagnosis of MS [27]. The disorders most often mistaken for MS have changed over time as a result of revisions to the diagnostic criteria for MS [28]. Non-specific white matter abnormalities on MRI, non-specific neurological symptoms, and small vessel ischemic disease are the most frequently reported findings in misdiagnosed patients [24].

MS is one of the most common neurological disorders that affects young adults (primarily female), and is highest on the differential if MRI demonstrates white matter lesions. FD patients may have (1) peripheral nerve symptoms that manifest as acute attacks of neuropathic pain in the limbs, especially under conditions of stress, heat, or fatigue [29]; and (2) cerebrovascular disease that affects both large and small vessels and can lead to the development of chronic white matter hyperintensities detected on brain MRI [5]. These features are consistent with MS, particularly in young and/or female patients who may have mild symptoms due to the progressive onset of FD and/or random X inactivation [30]. In these patients, neurological symptoms may be the first or only evidence of FD [31]. Cerebral small vessel involvement in FD patients may be due to endothelial cell dysfunction and deposition of neutral glycosphingolipids. Although the pattern of CWML in FD demonstrates a symmetric distribution frequently referred to as “vascular leukodystrophy”, variability in appearance due to aging and the temporal lesion load can confound the differential diagnosis [32]. Nevertheless, spinal cord involvement with characteristic neuroradiological findings, when present, is an additional powerful diagnostic element in MS [33]. Usually a careful neuroradiological analysis should be able to distinguish between white matter lesions that are highly suggestive of inflammatory events and MS, from those that are more typical of vasculopathy and FD [33].

Previous studies have identified subjects with a previous diagnosis of MS among patients with genetically proven FD [7, 8]. Conversely, in our study we identified FD subjects among patients who had initially received a “presumptive/possible” diagnosis of MS. We investigated a cohort of 86 individuals and identified four female patients with FD (4.7%). Three out of four patients had relatives previously diagnosed with FD (Patients 1, 2, and 3).

Our evaluation of the four affected patients indicated that family history and neurological signs are critical for the diagnosis of FD. Clinical manifestations in different organs including the kidney, heart, and eye should also be evaluated to support the FD diagnosis, particularly in male patients. FD should be considered in all cases of presumptive MS with atypical clinical presentation, atypical MRI findings, and the absence of oligoclonal bands in cerebrospinal fluid. It should also be considered if there is a family history of FD or clinical manifestations that could be attributed to this disorder. The ability to distinguish FD from MS is critical for the selection of the appropriate treatment.

## MATERIALS AND METHODS

### Patients

Blood was collected from 86 patients (58 women and 28 men) with a presumptive diagnosis of MS using EDTA as an anticoagulant. The study was approved by the Hospital Ethics Committee of the University of Palermo. Written informed consent was obtained from all participants. All patients were assessed by neurologists practicing in different neurological units in Italy.

### Genetic analysis

DNA samples were isolated from whole blood by column extraction (GenElute Blood Genomic DNA Kit, Miniprep, Sigma-Aldrich, USA). DNA concentrations were estimated using a spectrophotometer. Eight pairs of primers were designed to analyze eight target regions containing the seven exons of the *GLA* gene, including the flanking regulatory sequences, and the cryptic exon. PCR products were purified and sequenced using an automated DNA sequencer at BMR Genomics to identify mutations.

### Assays of α-gal A activity

We performed α-gal A activity assays on samples collected from male and female patients with positive *GLA* tests using the Dried Blood Filter Paper (DBFP) test described by Chamoles *et al*. [34] with minor modifications [35].

## Abbreviations

fD: Fabry disease
α-gal A: α-galactosidase A
CNS: central nervous system
CWML: chronic white matter lesions
MS: multiple sclerosis
MRI: magnetic resonance imaging
EDTA: ethylenediaminetetraacetic acid.
